# Fractionated radiation exposure amplifies the radioresistant nature of prostate cancer cells

**DOI:** 10.1038/srep34796

**Published:** 2016-10-05

**Authors:** N. McDermott, A. Meunier, B. Mooney, G. Nortey, C. Hernandez, S. Hurley, N. Lynam-Lennon, S. H. Barsoom, K. J. Bowman, B. Marples, G. D. D. Jones, L. Marignol

**Affiliations:** 1 Radiobiology and Molecular Oncology, Applied Radiation Therapy Trinity, Discipline of Radiation Therapy, Institute of Molecular Medicine, Trinity College Dublin, Ireland; 2 Department of International Health, Mount Sinai School of Medicine, New York, USA; 3 Molecular Medicine, Department Clinical Medicine, Trinity College Dublin, Ireland; 4 Department of Surgery, Trinity College Dublin, Ireland; 5 Department of Cancer Studies & Cancer Research UK Leicester Centre, University of Leicester, UK; 6 Department of Radiation Oncology, William Beaumont Hospital, 3811 W. Thirteen Mile Road, Royal Oak, MI 48073, USA

## Abstract

The risk of recurrence following radiation therapy remains high for a significant number of prostate cancer patients. The development of *in vitro* isogenic models of radioresistance through exposure to fractionated radiation is an increasingly used approach to investigate the mechanisms of radioresistance in cancer cells and help guide improvements in radiotherapy standards. We treated 22Rv1 prostate cancer cells with fractionated 2 Gy radiation to a cumulative total dose of 60 Gy. This process selected for 22Rv1-cells with increased clonogenic survival following subsequent radiation exposure but increased sensitivity to Docetaxel. This RR-22Rv1 cell line was enriched in S-phase cells, less susceptible to DNA damage, radiation-induced apoptosis and acquired enhanced migration potential, when compared to wild type and aged matched control 22Rv1 cells. The selection of radioresistant cancer cells during fractionated radiation therapy may have implications in the development and administration of future targeted therapy in conjunction with radiation therapy.

Following a prostate cancer diagnosis, approximately 50 percent of men will receive radiation therapy. Patients with PSA >20 ng/ml or biopsy Gleason score 8–10 or T2-3N0M0 localised prostate carcinoma are recognised as high risk^1^. The optimal management of these patients remains unclear. Randomized control trials recommend the combination of external beam radiotherapy with androgen deprivation therapy to improve overall survival^2^, but recurrence rates in these patients remain high and are associated with a limited chance of cure^3^. The characterisation of the radiobiological properties of prostate tumours, increasingly related to the eight cancer hallsmarks^4^, is essential to guide the evaluation of current as well as novel therapeutic options. It may also provide a means to select patients most likely to benefit from these strategies.

Modifications in the radiobiological properties of tumours can take several forms. Most likely, it results in an increased capacity of irradiated clonogens to overcome the anti-proliferative effects of radiation, evidenced by a quantifiable change in the relationship between clonogenic survival and radiation dose^5^. This change can be attributed to the capacity for these cells to overcome the induction and repair radiation damage^6^, ignore pro-apoptotic signals^7^ and avoid the transition to a senescent state^8^,^9^. But other factors complicate this relationship. First, tumour microenvironmental factors and the tumour vasculature^10^ may also reduce response to radiotherapy^11^. Second, rapidly accumulating evidence identifies the number of uncontrolled cancer stem cells following a radiotherapy regimen as a key to local tumour control probability^12^,^13^,^14^.

Exposure of cancer cells to fractionated radiation schedules can select a cancer subpopulation with modified cell fate in response to subsequent radiation exposure and affect tumour control probablity^15^. This selection process is increasingly reproduced *in vitro* to investigate the molecular response of cancer cells and guide the development of novel biomarkers of radiotherapy failure (reviewed in^16^). Few of these isogenic models currently exist for prostate cancer.

This study aimed to generate and characterise an isogenic model of radioresistant prostate cancer. Of the four commonly used prostate cancer cell lines, only 22Rv1 cells may be representative of primary disease^17^. This non-metastatic prostate cancer cell line was exposed to a fractionated radiation protocol. The resulting subline was evaluated for modification in radiation response and oncogenic properties. Our data suggests that this newly established radioresistant model has the potential to support discovery of novel biomarkers predictive of radiotherapy success.

## Results

### Selection of radioresistant 22Rv1 cells by fractionated irradiation

**Figure 1:**
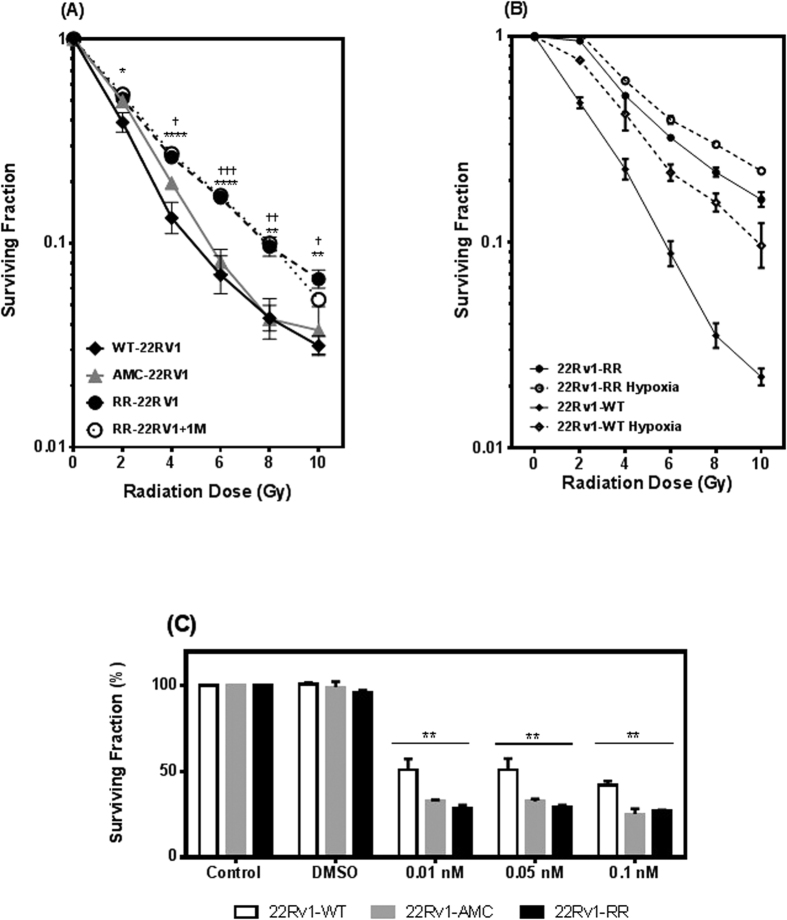
Generation of a stable isogenic radiation resistant prostate cancer cell line. (**A**) Surviving fraction, after 60Gy cumulative dose of X-radiation, of the radiation resistant (RR-22RV1) isogenic cell line model compared with 22RV1 wild type (WT-22RV1) control and 22RV1 age matched control (AMC-22RV1). The isogenic cell line shows consistent resistance to radiation after one month of growth with no treatment (RR-22RV1 + 1M). Survival of irradiated cells was measured by clonogenic cell survival assay after X-radiation with 0–10 Gy. Points are measured as means ± SEM of at least five individual experiments. (**B**) Surviving fraction of aerobic and hypoxic (0.5% O2, 24 hrs) WT-22Rv1 and RR-22Rv1 cells. (**C**) Clonogenic survival of 22RV1-WT, 2RRv1-AMC and 22RV1-RR cells treated with 0.01, 0.5 and 0.1 nM Docetaxel for 48 hours. Points are measured as means ± SEM of at least five individual experiments. Mean ± SEM, *refers to significant differences in the RR cell line vs WT control. ^†^refers to significant differences between RR and AMC cells. Δ refers to significant differences between AMC and WT control cells. *P ≤ 0.05, **P ≤ 0.01.

Wild type 22Rv1 (WT-22Rv1) were either exposed to 2-Gy fractionated radiation to a cumulative dose of 60Gy (RR-22Rv1) or mock irradiated (age matched controls AMC-22Rv1). At the end of this process, the proliferative potential following increasing radiation doses (2–10Gy) was measured in RR-22Rv1, AMC-22Rv1 and WT-22Rv1 using clonogenic assays. The individual experiments were used to define an average survival curve, with the deviation defined by summing the variance for each individual experiment at the corresponding dose and taking the square root (Fig. 1A). The surviving fraction of RR-22Rv1 cells was significantly higher than that of the WT-22Rv1 cell line at all doses tested. This increase was sustained one month later (RR-22Rv1-1M). Each experimental repeat was fitted with a linear curve between 0–6 Gy. The slope was then calculated to assess the decrease in survival. Wild-type 22RV1 cell survival decreased 15.24%/Gy (±0.6%), age-matched control cell survival decreased 15.20%/Gy (±0.37%), and radiation resistant cell survival decreased 13.72%/Gy ± (0.38%). There was no significant difference in the survival of wild type and age-matched controls (p > 0.05), but there was a significant difference between wild type and radiation resistant (p < 0.001) and between age-matched control and radiation resistant (p < 0.001) cells. The selection of radioresistant 22Rv1 cells was further evidenced by an increase in the area under the curve of the radiation survival curve from 2.3 (WT-22Rv1) to 3.14 (RR-22Rv1 cells). Mock-irradiated, aged-matched controls (AMC-22Rv1) exhibited an intermediate area under the curve of 2.7 and significantly reduced clonogenic survival compared to that of RR-22Rv1 cells at all doses tested, but 2 Gy (p > 0.05).

### Hypoxic response of selected radioresistant 22Rv1 cells

To evaluate whether the radioresistance of RR-22Rv1 could be further increased, the clonogenic survival of WT-22Rv1 and RR-22Rv1 was measured following exposure to 0.5% oxygen for 24 h prior to irradiation (Fig. 1B). Hypoxic treatment increased the survival of WT-22Rv1 cells by up to 4-fold following irradiation (ANOVA, p < 0.0001) with an oxygen enhancement ratio of ~2.2 at 50% survival. The clonogenic survival of RR-22Rv1 cells was significantly higher than that of hypoxic WT-22Rv1 cells over the 0–6 Gy dose range (mean difference in survival of 18% at 2 Gy (p < 0.001), 10% at 4 Gy (p < 0.05) and 11% at 6 Gy (p < 0.05)). Hypoxia further protected RR-22Rv1 from the effect of radiation with a significant increase in clonogenic survival at all doses tested with an oxygen enhancement ratio of ~1.2.

### Sensitivity of selected radioresistant 22Rv1 cells to Docetaxel

The response of RR-22Rv1, AMC-22RV1 and WT-22RV1 cells to docetaxel was investigated using clonogenic assays (Fig. 1C). Clonogenic survivals following docetaxel treatment (0.01, 0.05, 0,1nM, 48 h) were significantly reduced in 22Rv1-RR and 22Rv1-AMC, when compared to 22Rv1-WT cells at all concentrations tested (p < 0.005).

### S-phase cell cycle fraction of radioresistant 22Rv1 cells

**Figure 2:**
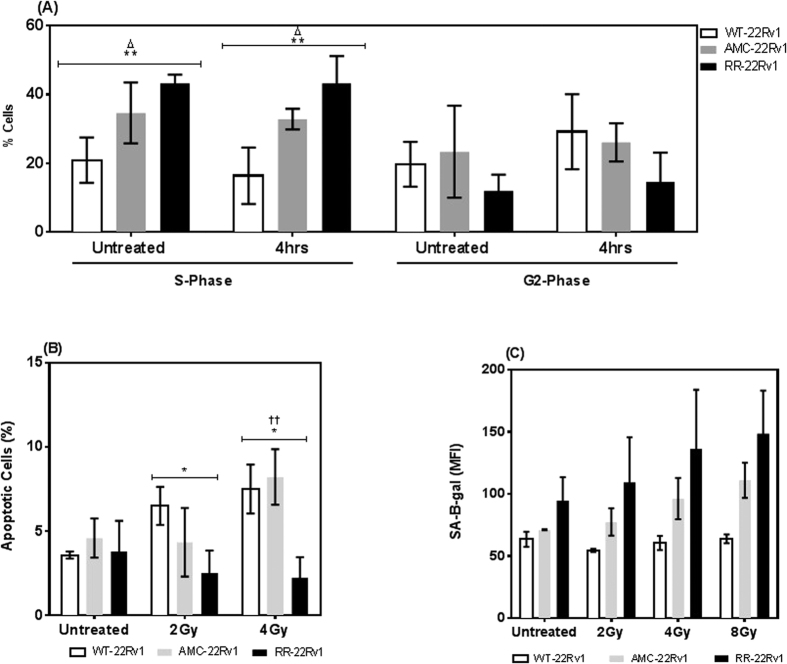
Fractionated radiation exposure selects for 22Rv1 cells with enriched S-phase fraction and apoptosis resistance. (**A**) Cell cycle analysis of WT-22RV1, RR-22RV1 and AMC-22RV1 cells untreated and four hours post irradiation with 2 Gy. (**B**) Radiation induced apoptosis measured using Annexin V and PI staining in WT-22RV1, RR-22RV1 and AMC-22RV1, 6 hours after treatment with 2 Gy and 4 Gy X-irradiation. (**C**) SA-*β*-gal activity measured with C_12_FDG and flow cytometry, with and without treatment of 2 Gy, 4 Gy and 8 Gy of ionising radiation. n = 2 *refers to significant differences in the RR cell line vs WT control. ^†^refers to significant differences between RR and AMC cells. Δrefers to significant differences between AMC and WT control cells. *P ≤ 0.05, **P ≤ 0.01.

Cells cycle distributions were next measured by PI staining and flow cytometry (Fig. 2A). The % of cells in the S-phase was significantly higher in RR-2Rv1 (43.2 ± 1.3%) than in WT-22Rv1 (20.9 ± 3.3%, p = 0.0007) but not AMC-22Rv1 (34.6 ± 5.1%, p = 0.11). This pattern was maintained following irradiation (2-Gy) (ANOVA, p = 0.002). Radiation exposure did not increase the % of S-phase cells in all three lines (WT-22Rv1, p = 0.42; AMC-22Rv1, p = 0.76; RR-22Rv1, p = 0.99). The % of cells in the G2 phase were not significant different in both untreated (ANOVA, p = 0.23) and irradiated cell lines (ANOVA, p = 0.11).

### Apoptosis sensitivity of radioresistant 22Rv1 cells

Apoptotic cell death following exposure to radiation (2, 4 Gy) was quantified in all three lines using annexin V-FITC/PI staining and flow cytometry and compared to that of aged matched controls (Fig. 2B). The % of cells in early apoptosis was significantly reduced in RR-22Rv1 when compared to WT-22Rv1 and AMC-22Rv1 at both doses. The baseline % of apoptotic cells were similar across the three cell lines (ANOVA, p = 0.77). Following exposure to radiation (2, 4 Gy), the % of apoptotic cells increased in WT-22Rv1 (2 Gy: 3.58 ± 0.15 to 6.50 ± 0.65. p =  0.024; 4G y: 3.58 ± 0.15 to 7.5 ± 0.83, p = 0.03) and AMC-22Rv1 (2 Gy: 4.59 ± 0.67 to 4.34 ± 1.17, p = 0.86; 4 Gy: 4.59 ± 0.67 to 8.22 ± 0.95, p = 0.03) but not in RR-22Rv1 cells (2 Gy: 3.79 ± 1.05 to 2.51 ± 0.77, p = 0.38; 4 Gy: 3.79 ± 1.05 to 2.25 ± 0.7, p = 0.28).

### Susceptibility to radiation-induced senescence of radioresistant 22Rv1 cells

The levels of senescence-associated β-galactosidase (SA-β-Gal) were next measured by flow cytometry^18^ (Fig. 2C). A non-significant trend towards elevated β-galactosidase levels was seen in untreated RR-22Rv1 cells, when compared to AMC- and WT-22Rv1 cells (ANOVA, p = 0.14). A similar trend was observed in irradiated cell lines (ANOVA, 2 Gy, p = 0.18; 4 Gy p = 0.17, 8 Gy p = 0.06). Radiation exposure appeared to increase senescence levels within each of the three lines, but the differences did not reach statistical significance (ANOVA, WT-22Rv1 p =  0.32. AMC-22Rv1, p  = 0.22. RR-22Rv1, p = 0.56).

### DNA repair capacity of radioresistant 22Rv1 cells

**Figure 3:**
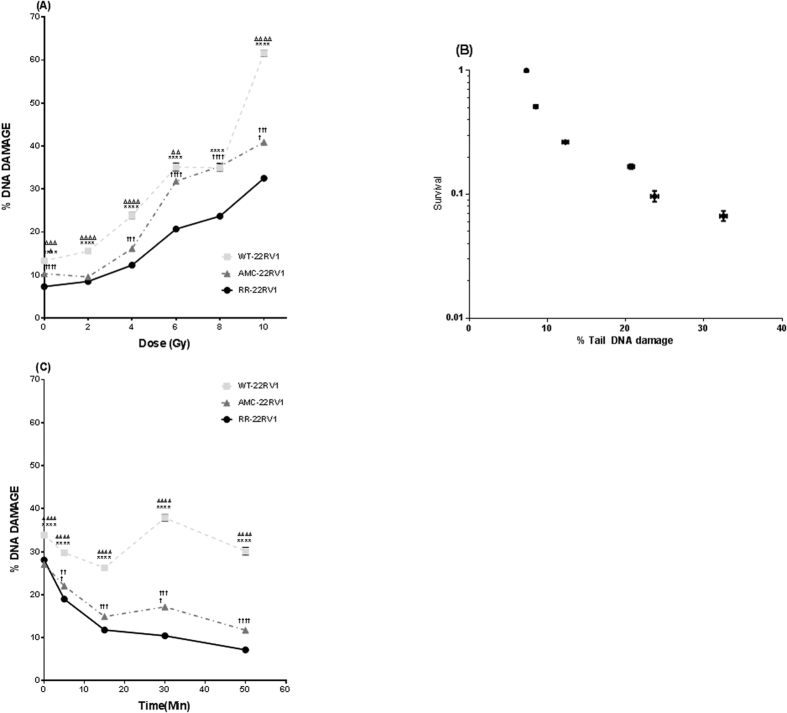
Increased proliferative potential following radiation exposure is associated with reduced levels of DNA damage. (**A**) DNA damage in response to treatment with a range of radiation doses (0–10 Gy) in each of the three cell lines as measured by alkaline comet assay. N = 3 independent experiments. (**B**) Correlation between clonogenic survival and % tail DNA damage in 22Rv1-RR cells. (**C**) DNA repair capabilities of the 22Rv1-RR, WT and AMC cell lines. Initial damage and repair up to 50 min post irradiation with 8 Gy is measured using the alkaline comet assay. n = 5 independent experiments. DNA damage was calculated using % tail DNA for each comet, 400 comets were scored on two slides/4 gels per replicate and SEM calculated. *refers to significant differences in the RR cell line vs WT control. ^†^refers to significant differences between RR and AMC cells. Δ refers to significant differences between AMC and WT control cells. *P ≤ 0.05, **P ≤ 0.01.

The alkaline comet assay (ACA) was used to quantify immediate levels of induced DNA damage (% Tail DNA) in the three cell lines following irradiation (0–10 Gy) (Fig. 3A). The level of initial DNA damage was significantly lower in RR-22Rv1 cells when compared to both AMC-22Rv1 and WT-22Rv1 in unirradiated cells and sustained across all doses tested (ANOVA, p < 0.0001). Initial DNA damage formation increased exponentially with increasing radiation dose in all three lines. At 10 Gy, the absolute increase in % Tail DNA compared to unirradiated control was highest in 48.5 ± 0.9% in WT-22Rv1 (0 Gy: 13.3 ± 0.55% to 10 Gy 61.6 ± 0.7%) 30.6 ± 0.68% in AMC-22Rv1 (0 Gy: 10.3 ± 0.36% to 10 Gy: 40.9 ± 0.6%) and lowest in 25.47 ± 0.5% in RR-22Rv1 (0 Gy: 7.03 ± 0.32% to 10Gy: 32.5 ± 0.44%). A strong correlation between the % tail DNA and clonogenic survival was identified in all three lines (22Rv1-RR, r = −0.79, p = 0.0028; 22Rv1-AMC, r = −0.94, p = 0.01; 22Rv1-WT, r = −0.94, p = 0.01) (Fig. 3B). ACA was next used to assess the extent of DNA damage repair for up to 50 min following 8Gy exposure (Fig. 3C). The DNA damage levels decreased rapidly within the first 15 min in all three lines. The reduction was greatest in RR-22Rv1 (2.37-fold), when compared to AMC-22Rv1 (1.81-fold) and WT-22Rv1 (1.3-fold) (p < 0.0001). For RR-22Rv1, beyond this time point, the % amount of DNA damage continued to decrease, albeit at a slower pace. Fifty minutes post exposure, the initial % tail DNA was reduced 3.9-fold in RR-22Rv1 cells (p < 0.0001) compared to 2.3- (p < 0.0001) and 1.12-fold (p = 0.005) in AMC-22Rv1 and WT-22Rv1 cells, respectively.

### ROS levels in radioresistant 22Rv1 cells

**Figure 4:**
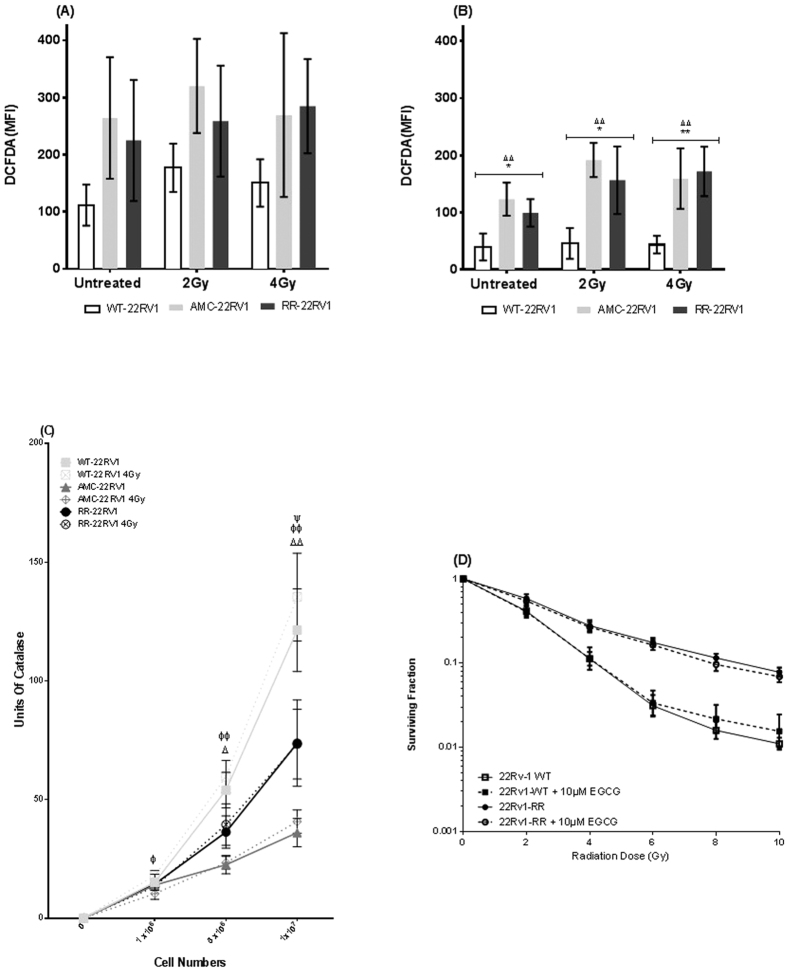
Fractionated radiation exposure selects for cells with high reactive oxygen species levels. (**A**) Reactive Oxygen species levels in the live cell population (isolated using PI) following treatment with radiation (2/4 Gy) compared to untreated controls as measured by Median Fluorescence Intensity (MFI) of DCFDA. Mean ± SD for 4 experiments. (**B**) Levels of reactive oxygen species in the measured in the dead/dying cell population (isolated using PI uptake) following treatment with radiation (2/4 Gy) compared to untreated controls as measured by Median Fluorescence Intensity (MFI) of DCFDA. Mean ± SD for 4 experiments. (**C**) Catalase activity of isogenic radiation resistant cell lines and controls before and after treatment with 4 Gy x-irradiation. Points mean of 4 independent experiments ± SEM. (**D**) Radiation clonogenic survival curve of 22Rv1-WT and 22Rv1-RR cells treated with 10 uM ECGC and untreated controls. Mean ± SD for 4 experiments. *refers to significant differences in the RR cell line vs WT control, ^†^refers to significant differences between RR and AMC cell lines and Δ refers to significant differences between AMC and WT control cells. Φ differences between WT 4 Gy vs AMC 4 GY, ψ differences between WT 4 Gy and RR 4 Gy. *P ≤ 0.05, **P ≤ 0.01, ***P ≤ 0.001, ****P ≤ 0.0001.

The levels of reactive oxygen species (ROS) in the live and dead cell subpopulations of irradiated (2 Gy, 4 Gy) and unirradiated cells were measured using CM-H_2_DCFDA staining analysed by flow cytometry. In the live cells (Fig. 4A), the measures of ROS appeared elevated in RR-22Rv1 and AMC-22Rv1 cells but were not significantly different between the three cell lines for the unirradiated (ANOVA, p = 0.092) and the irradiated cells (ANOVA, 2 Gy, p = 0.07; 4 Gy, p = 0.16). Similarly, exposure to radiation did not significantly change the amounts of ROS within each of the three lines (ANOVA, WT-22Rv1, p = 0.12 ; AMC-22Rv1, p = 0.74, RR-22Rv1, p = 0.68). In the dead/dying subpopulation of cells (Fig. 4B), the measures of ROS were significantly different between the three cell lines in the unirradiated (ANOVA, p = 0.003) and irradiated cells (ANOVA, 2 Gy, p = 0.001; 4 Gy, p = 0.002) and highest in the AMC-22Rv1 and RR-22Rv1 cells. However, exposure to radiation did not significantly change the levels of ROS within all three lines (ANOVA, WT-22Rv1, p = 0.92; AMC-22Rv1, p = 0.09, RR-22Rv1, p = 0.10). Catalase activity was measured in irradiated (4 Gy) and unirradiated cells (Fig. 4C). At a cell number of 5 × 10^6^, catalase activity was significantly different between the three cell lines in unirradiated (ANOVA, p = 0.01) and irradiated cells (ANOVA, p = 0.01). Subgroup comparisons indicate that catalase activity is significantly lower in AMC-22Rv1 cells, when compared to WT-22Rv1 under both conditions. In response to radiation exposure, catalase activity was not statistically different in WT-22Rv1 (p = 0.5), AMC-22Rv1 (p = 0.85) and RR-22Rv1 (p = 0.78), when compared to unirradiated controls. Finally, the radiation clonogenic survival curve of both WT-22Rv1 and RR-22-Rv1 cells was not modified following treatment with the anti-oxidant epigallocatechin-3-gallate (EGCG) (Fig. 4D).

### Migration capacity of 22Rv1 cells

**Figure 5:**
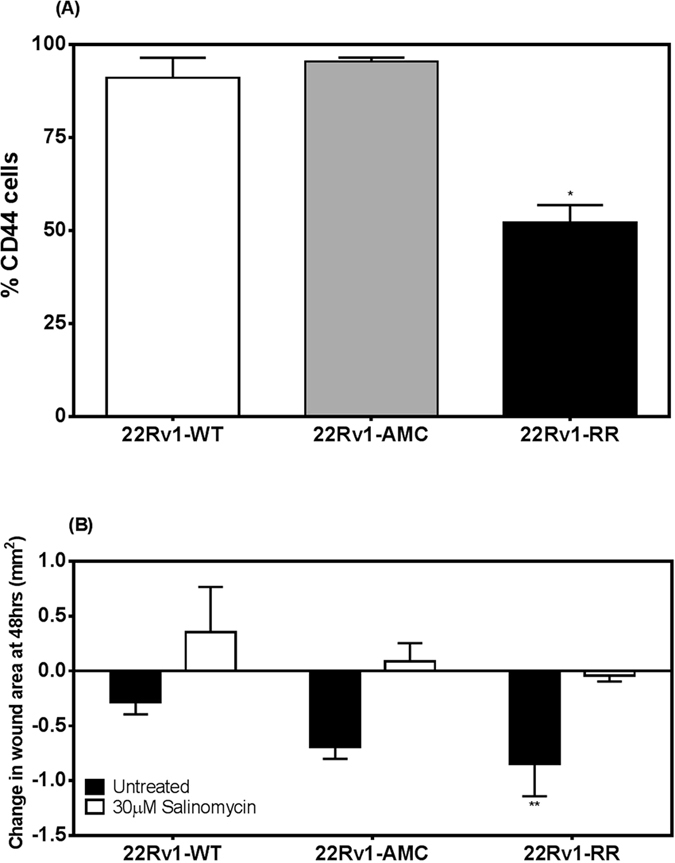
Exposure to fractionated radiation selects for 22Rv1 cells with reduced CD44 expression and enhanced migration capacity. (**A**) The %CD44 positive cells was determined by flow-cytometry from the fluorescence intensities measured in 22Rv1-RR, AMC, and WT cells stained with FITC-conjugated CD44 antibody (**B**) The change in wound area was measured 48 hours following injury in 22Rv1-WT, AMC and RR cells in the absence or presence of 30 μM salinomycin (24 hours). n = 3; *p < 0.05.

The CD44+ fraction was determined in each cell line by flow cytometry (Fig. 5A). The fraction of CD44+ cells was significantly reduced in the radioresistant RR-22RV1 (52%), when compared to WT-22RV1(91%) and AMC-22RV1 cell populations (95%) (ANOVA, p < 0.0001). There was no significant difference between the fractions of CD44+ cells in the WT-22RV1 cell population when compared to the AMC-22RV1. A wound healing assay was performed on RR-22Rv1, AMC-22Rv1 and WT-22Rv1 cells to assess cellular motility (Fig. 5B)^19^. Over a 48 h period, wound healing was most pronounced in 22Rv1-RR, as evidenced with significantly decreased wound area, when compared to 22Rv1-WT cells (p = 0.02). This effect was prevented by treatment with the stem cell inhibitor salinomycin in all three lines (Fig. 5B).

## Discussion

Isogenic models of radioresistance have been generated through exposure of cancer cell lines to a variety of fractionation schedules with total doses within a 40–60 Gy range^20^,^21^,^22^ and overall treatment times varying from 5 days^23^ to 6 years^16^,^24^. In prostate cancer, isogenic models of LncaP, PC-3 and DU145 cells were generated through 2 Gy daily exposure over 5 consecutive days and associated with a 1.6, 1.5 and 1.5 fold increase in the radiation dose needed to induce 0.1% survival (dose modifying factor), when compared to wild type cells^23^. The radiation-surviving cell population of DU145, PC-3, LnCaP and 22Rv1 cells following exposure to 35 doses of 2 Gy was isolated to examine effects on plasticity^25^ and neuroendocrine differentiation^26^. A number of limitations prevent the reproduction of a clinical radiotherapy delivery under experimental conditions such as cell ageing and the necessity for recovery periods^16^.

In this study, 22Rv1 cells were exposed to repeated 2 Gy-dose fractions and allowed to recover to a set confluence of ~70–80% in between fractions. The cumulative exposure of 22Rv1 cells to 60 Gy-fractionated radiation resulted in the generation of a sub-line with a significantly increased clonogenic survival potential following radiation exposure, when compared to wild type and mock irradiated, aged-matched cells. Increased colony forming ability is one of the most commonly reported consequences of protracted fractionated radiation exposure and is often associated with a modification of cell cycle distribution^16^,^22^. The resulting RR-22Rv1 cell line was enriched in S-phase cells, when compared to WT-22Rv1 cells. Enrichment in this radioresistant cell cycle phase has been reported in other radioresistant cell models^16^. In prostate cancer specimens, a larger S-phase fraction has been associated with more aggressive tumours^27^,^28^ and reduced local tumour control probability following radiotherapy^29^. Further evaluation of the underlying mechanisms for the amplification of the S-phase cell population is warranted.

The microtubule targeting agent Docetaxel is the standard of care first line chemotherapeutic drug for the treatment of hormone refractory prostate cancer^30^. Its effect is however limited by the poorly understood development of taxane-refractory tumours^31^. RR-22Rv1 and AMC-22Rv1 cells were more sensitive to Docetaxel than WT-22Rv1, suggesting distinct, possibly age-related, mechanisms of resistance.

Evaluations of the volume of prostate tumours suggest that 20% is exposed to less than 5 mmHg (0.7%) oxygen and prostate tumours are therefore considered hypoxic in nature^32^. Clinical evidence and modelling studies have demonstrated that tumour cell proliferation rates, DNA repair capacity and cellular hypoxia collectively modulate tumour control probability in response to radiation therapy^33^. The resulting RR-22Rv1 line was significantly more resistant to radiation than hypoxic WT-22Rv1 cells. Further enhancement of the radioresistance of RR-22Rv1 cells by hypoxia was small, suggesting that levels of radioresistance may have peaked in these cells.

Formation of DNA damage is the key event for cell killing by ionizing radiation^34^ and a cell’s ability to repair such DNA damage is key to determining a cell’s fate following irradiation^35^. Analysis of % Tail DNA immediately and up to 50 min after radiation exposure indicated that RR-22Rv1 cells were significantly less sensitive to initial damage induction than WT-22Rv1 and AMC-22Rv1 cells and repaired damage more effectively. This characteristic of radioresistant cells was documented in other isogenic cell models^16^,^36^,^37^. In prostate cancer patients receiving androgen deprivation therapy, the induction of reduced DNA repair capacity was proposed as a possible underlying mechanism for the improved response of these patients to subsequent radiotherapy treatment^38^. The % tail DNA damage peaked at 30 min post-irradiation in WT-22Rv1 cells but not in AMC-22Rv1 or RR-22Rv1 cells. The mechanistic interpretation of this peak should be further investigated. It may reflect induction of DNA damage by secondary radiation-induced reactive oxygen species, such as those released by the mitochondria^39^.

Mitochondrial respiration is increasingly documented to contribute to tumor cell survival and proliferation^40^. Mitochondrial dysfunction has been associated with cellular fate following irradiation^41^ and a direct role in radiation-induced G2/M arrest^39^ and apoptosis induction has been proposed^42^. Following irradiation, WT-22Rv1 and AMC-22Rv1 cells were more susceptible to apoptosis induction than RR-22Rv1 cells. Modification in radiation-induced apoptosis sensitivity has been reported in some^43^ but not other^21^ isogenic models. The prognostic potential of apoptosis sensitivity in prostate radiotherapy patients has been proposed^44^ but remains unconfirmed. Nonetheless, the pharmacologic radio sensitization of prostate cancer cells is often achieved through induction of apoptosis^45^. Further evaluation of the underlying mechanisms for this reduced susceptibility to radiation-induced apoptosis is required.

The induction of permanent growth arrest through senescence was proposed as an alternative mechanism in the therapeutic response to therapy in cancer cells with deregulated apoptotic signalling^46^. The induction of senescence in response to elevated DNA damage has been associated with increased therapeutic sensitivity^47^. In prostate cancer patients, increased expression of β-galactosidase (GLB1) was associated with a reduced risk of recurrence^48^. A proportion of surviving DU145 and PC3 cells exposed to 35 fractions of 2 Gy developed a senescent-like morphology associated with elevated mRNA expression of key senescence markers^25^. Our RR-22Rv1 cells however failed to show statistically significant evidence for a possible reduction in the induction of therapeutic senescence. Our analysis of β-galactosidase activity would not detect other permanent growth arrests such as the induction of neuroendocrine differentiation (NED). Therapeutic NED has also been identified as a cause of radioresistance that may be limited to CD44+ prostate cancer stem cells^49^. The evaluation of the stemness of our model is pending, but our preliminary analysis of CD44 expression suggests that exposure to fractionated radiation reduces the fraction of CD44-expressing 22Rv1 cells. Downregulation of CD44 was associated with increased radiosensitivity in DU145 and PC3 cells^50^, but increased migration potential^51^. Wound closure was more efficient in CD44-deprived RR-22Rv1 than WT-22Rv1 or AMC-22Rv1 cells. This process was inhibited by salinomycin, an anti-coccidial drug increasingly tested for its potential anti-cancer and anti-cancer stem cell properties^52^,^53^. This sodium ionophore monensin similarly inhibited proliferation and migration of LnCaP cells^51^. Further investigation into the role of CD44 in the selection of a radioresistant subpopulation of cells is warranted.

The ability of prostate cancer cells to manage oxidative stress plays an important role in cell signalling and their response to therapeutic injury^54^. In particular, reduced susceptibility to ROS-induced cellular damage and efficient repair of radiation-induced DNA damage was proposed as an underlying mechanisms to the observed radioresistance of prostate cancer stem cells^55^. Lower ROS basal levels were associated with increased Nfr2 levels and radiosensitivity in prostate cancer cells^56^. Basal ROS levels were lower in WT-22Rv1, than in AMC-22Rv1 and RR-22Rv1 cells. This reduction was associated with increased catalase activity in WT-22Rv1 cells. ROS levels were elevated in dying RR-22Rv1 cells. This suggests that cells with high ROS levels are preferentially eliminated in this cell population or reflects the likely release of ROS in dying cells. However, the sensitivity of cells to the anti-oxidant epigallocatechin-3-gallate (EGCG), whose therapeutic potential in prostate cancer was proposed previously^57^, remained unchanged in both WT- and RR-22Rv1 cells.

## Conclusion

Exposure to fractionated radiation progressively selected for 22Rv1 cells with enhanced oncogenic properties protective against radiation injury and supportive of tumour invasion. The characterisation of the radioresistance of these cells using *in vivo* dose response assays is required to account for the likely effect of spontaneous (non-radiation) death rates^58^ and the tumour microenvironment^59^ on tumour control probability in a clinical setting^15^. This data must be considered within the context of one cell line, and the limitations of *in vitro* models, but the phenotyping modifications observed support the clinical relevance of this model to enable further study of the mechanisms of radioresistance in prostate cancer cells.

The progressive selection of radioresistant cells throughout a protracted radiation therapy protocol may have clinical implications. In the era of personalised medicine, concurrent targeted therapies may require careful timing and perhaps may be more effective when administered toward the end of radiotherapy delivery.

## Materials and Methods

### Cell lines and culture

The human prostate cancer cell line 22Rv1 was obtained from American Type Culture Collection. Cells were maintained as monolayers in RPMI cell culture medium containing l-glutamine (Lonza, Castleford, UK) with 10% foetal bovine serum (Gibco, Dublin, Ireland) and 1% pen/strep (Lonza). The cells were maintained at 37 °C in 95% humidified air containing 5% CO_2_ and sub-cultured once to twice weekly to maintain exponential growth unless otherwise stated. Cells were grown to approximately 70–80% confluency in vented 75 cm^2^ culture flasks prior to irradiation. Each irradiation consisted of 2 Gy X-rays dose (250 keV, 15 mA) using an RS225 cabinet irradiator (Gulmay Medical, Surrey, UK). This process was repeated weekly until the cells received to a total cumulative of 60 Gy. Mock irradiated cells were cultured in parallel as age-matched controls (AMC-22Rv1). Hypoxia (0.5% O_2_, pO_2_ < 2 mmHq) was achieved by exposing cells in a 1000 *in vivo* hypoxic chamber (BioTrace, Bracknell, UK) to a mixture of nitrogen, CO_2_ (5%) and compressed air to achieve a 0.5% oxygen concentration.

### Clonogenic Assay

Cell survival was evaluated using a standard colony forming assay. Cells in exponential growth phase (approx. 70% confluent) were harvested by trypsinisation (Lonza) and counted in order to obtain a single cell suspension of 1 × 10^6^ cells/ml. Cells were seeded into six well plates at a density of 1 × 10^3^ −1 × 10^4^ cells/well and allowed to adhere to plates overnight in the incubator at 37 °C in 95% humidified air containing 5% CO_2_. Cells were then irradiated (2, 4, 6, 8 or 10 Gy single doses) and incubated for 7–10 days under the conditions previously described. Following incubation, colonies were fixed and stained using 0.05% crystal violet in 70% methanol solution. Plates were dried overnight and colonies containing >50 cells counted using the ColCount instrument (Oxford Optronix Ltd, Oxford, UK). Surviving fraction was calculated as no. colonies/(no. cells seeded X PE). The platting efficiency PE was calculated using the no. colonies/no. cells seeded in the unirradiated cells.

### Cell Cycle Analysis

Cell cycle analysis was performed using Propidium Iodide (PI) staining and flow cytometry as previously described^60^. Cells were harvested at 4 h, 8 h, 12 h, 24 h, 30 h post irradiation, using trypsin-EDTA(Lonza), and the pellet fixed and permeabilised by dropwise addition of 4 ml ice cold 70% EtOH (Sigma-Aldrich, Wicklow, Ireland). Cells were fixed for a minimum of 2 h at 4 °C. Fixed cells were centrifuged at 180 g × 3 min and the supernatant decanted, samples were then washed in PBS and centrifuged as before. Each sample was resuspended in 0.5 ml Triton X-100 (0.1% v/v in PBS)(Sigma-Aldrich) containing PI (0.02 mg/ml PI)(Sigma-Aldrich) and RNase A (0.2 mg/ml) (Sigma-Aldrich). Unstained fixed controls were resuspended in PBS. Samples were incubated at 37 °C for 30 min and analysed immediately using CyAn^TM^ ADP Analyzer (Beckman Coulter, Clare, Ireland.). Cell cycle phase was determined using flowJo software (FlowJo LLC, Or, USA).

### Apoptosis Assay

Apoptotic cells were detected using annexin V-FITC/PI staining and flow cytometry as previously described^61^. Following treatment with 0, 2 or 4 Gy of X-irradiation, cells were trypsinised and combined with supernatant containing non-adherent cells. Cell pellets were resuspended in ice cold 1X binding buffer (0.1M Hepes (Sigma-Aldrich) 1.4M NaCl (Sigma-Aldrich) and 25 mM CaCl_2_, at pH 7.4 (Sigma-Aldrich). Cells were stained with 3 μl Annexin V-FITC (IQ Products, Groningen, Netherlands) and incubated at 4 °C. Cells were washed in ice cold PBS and resuspended in 1X binding buffer containing PI (0.05 μg/ml) (Invitrogen, Dublin, Ireland). Cell were analysed using the CyAn^TM^ ADP Analyser (Beckman Coulter) and apoptotic cells were detected using the FlowJo software (FLowJo LLC, Or, USA).

### Senescence

β-Galactosisdase activity was measured by flow cytometry as previously reported^18^. Cells were seeded in 6 well plates at a density of 1–2 × 10^5^ cells/well and allowed adhere overnight prior to irradiation (2 Gy, 4 Gy or 8 Gy) and returned to the incubator along with mock irradiated controls for 24 h. Cells were washed with PBS and incubated for 1 h at 37 °C, 5% CO_2_ with Bafilomycin A1 (Sigma-Aldrich) supplemented growth media to adjust lysosomal pH to pH6. 2 mM 5-Dodecanoylaminofluorescein di-β-D-galactopyranoside (C_12_FDG) (Sigma-Aldrich) was added and samples were incubated for 1 h before analysis. Cells were then trypsinised and resuspended in 1 ml PBS and analysed immediately using a CyAn^TM^ ADP Analyser (Beckman Coulter). Mean fluorescence intensity was calculated using FlowJo software.

### Alkaline comet assay

A previously reported version of the alkaline comet assay (ACA)^62^ adapted from the original protocol by Singh *et al*.^63^ was used to determine levels of DNA damage and repair capability after irradiation. All reagents used were obtained from Sigma-Aldrich, Dorset, UK. Briefly, 4 × 10^4^ cells resuspended in low melting point agar (0.6% in PBS) were layered onto agar-coated slides (1% in ddH_2_O) prior to irradiation on ice and in the dark. Following this slides for the repair study were incubated at 37 °C, 5% CO_2_, 95% humidified air in RPMI media containing 20% FBS and 5% pen/strep for up to 50 min. All slides were then incubated overnight in ice cold lysis buffer (100 mM NA_2_EDTA, 2.5M NaCL, 10 mM Tris-HCL, pH 10 with 1% Triton X added immediately before use). Following electrophoresis (30 V, 30 mA, 20 min in 300 mM NaOH, 1 mM Na_2_EDTA, pH 13), the slides were incubated in neutralization buffer (0.4M Tris-HCL, pH7.5), washed and allowed to dry for 2 h at 37 °C prior to PI staining (2.5 μg/ml). Comets were visualised and measured using Komet 5.0 software (Kinetics imaging software ltd, Brombourough UK) and fluorescent microscope at a magnification of 20X. 50 comets were scored/gel. The mean % tail DNA, calculated as the percentage of DNA present in the comet tail (measured as an intensity) compared to total comet intensity), was deduced from three (dose response) or four (repair study) independent experiments and presented as mean ± SEM.

### Oxidative Stress

Intracellular oxygen species were detected using Di(Acetoxymethyl Ester) (6-Carboxy-2′,7′-Dichlorodihydrofluorescein Diacetate) (CM-H_2_DCFDA)(Life Technologies, Dublin, Ireland) and flow cytometry. Exponentially growing cells were collected by trypsinisation. Samples were incubated for 30 min in the dark at 37 °C, 5% CO_2_, 95% humidified air in PBS containing 10 μM CM-H_2_DCFDA, with controls incubated in PBS. After incubation cells were pelleted at 180 × g for 3 min and the supernatant decanted. Samples were resuspended in PBS and irradiated with 2 Gy or 4 Gy X-rays, controls were mock irradiated and placed directly on ice. PI (1 mg/ml) was added to the samples except appropriate controls. Samples were analysed immediately using CyAn^TM^ ADP Analyser (Beckman Coulter). Data was analysed using FlowJo software.

### Catalase Activity

Catalase activity was measured using a visual method as previously reported^64^. Cells harvested as previously described were resuspended in pyrex tubes (Corning, Flintshire, UK) in varying concentrations (10^5^–1.5^7^ cells) in 100 μl of PBS (Lonza) prior to mock or 4-Gy irradiation. Catalase powder (Sigma-Aldrich) was dissolved in 100 μl distilled pure water to generate a calibration curve for catalase activity (0–200units). 100 μl1% Triton X-100 and 100 μl undiluted hydrogen peroxide (30%) were added to the solutions, mixed thoroughly and incubated at room temperature for 15 min before the height of O_2_-forming foam was measured with a ruler.

### Migration Assay

A wound healing assay was performed with the Ibidi Wound Healing 6 Well Plate Assay Kit (Ibidi, GmbH, Martinsried, Germany). Cells were seeded into Ibidi insert wells at 500,000 cells/well with total volume 70 μl, allowed to adhere for 24 h and incubated in 30 μM salinomycin (Sigma-Aldrich) treated media for 24 h. After incubation, the insert was removed and images were taken every at 24 and 48 h. Image analysis and migration rate determination was performed using ImageJ Analysis software (Bethesda, USA).

### CD44 Expression

Cell pellets were resuspended in staining buffer containing CD44 (FITC-conjugated mouse monoclonal anti-human, BD Pharminogen) and incubated for 20 min at 4 °C in the dark. Cells were washed and fluorescence intensity was measured using CyAn^TM^ ADP Analyser (Beckman Coulter).

### Docetaxel treatment

Cells were seeded into 6 well plates at a density of 750 cells/well for control and vehicle control (1X DMSO) and 3 × 10^3^ cells/well for treated. Cells were treated with 0.01 nM, 0.05 nM, 0.1 nM, 0.25 nM, 0.5 nM and 1 nM of Docetaxel (Sigma Aldrich) for 48 h or left untreated as controls.

### Statistical Analysis

All statistical analysis was carried out using Prism (version 6 for mac OS X and windows 7, Graphpad software La Jolla California USA, www.graphpad.com). The analysis of differences in the means of continuous data was performed with unpaired two tailed Student *t*-Tests, under the assumption that the data is Normally distributed. A One-way ANOVA with Bonferroni correction was used when the means of more than two groups were compared. A spearman correlation was used to quantify the non-linear relationship between the % tail DNA and clonogenic surviving fractions. All experiments were performed in triplicates unless otherwise stated. A *p*-value < 0.05 was considered statistically significant.

## Additional Information

**How to cite this article**: McDermott, N. *et al*. Fractionated radiation exposure amplifies the radioresistant nature of prostate cancer cells. *Sci. Rep*. **6**, 34796; doi: 10.1038/srep34796 (2016).
